# Important Considerations for ADHD ‘Patient and Public’ Involvement and Engagement in Research

**DOI:** 10.18103/mra.v11i10.4477

**Published:** 2023-10-25

**Authors:** B. French, A. Price, A. Salimi, A. Russell

**Affiliations:** 1 School of Psychology, University of Nottingham, Nottingham, UK; 2 School of Medicine, University of Nottingham, Nottingham, UK; 3 Health and Community Sciences, University of Exeter Medical School, Exeter, UK; 4 Department of Public Health and Sports Sciences, University of Exeter Medical School, Exeter, UK

## Abstract

In this letter, we summarise key points of learning from research projects on attention deficit hyperactivity disorder (ADHD) that have had patient and public involvement and engagement (PPIE) as a key part of the research process. We share learning from our experiences in delivering research working with PPIE groups with ADHD, as top tips for researchers. Our aim is to highlight the importance of including lived-experience in ADHD research, share learning and highlight some of the (potentially invisible) differences in functioning that someone with ADHD can experience in relation to attentional focus, organisation, and time management. Specifically, how these might impact working practices of PPIE groups that include people with ADHD.

## What is PPIE? The Importance of Including Stakeholders Throughout Research Cycle

Patient and public involvement and engagement or PPIE is a formal term for research projects (mainly in the area of health) that involve members of the public in all stages of the research process, alongside academic researchers. PPIE can inform all aspects of the research cycle^1^ such as:

∘ which questions are researched
∘ how the research project is conducted (e.g. how people are recruited, what information they have to decide whether to participate)
∘ what specific research tasks participants are asked to do (e.g. questionnaires, surveys, experiments)
∘ how the data are analysed and interpreted o what the findings and implications of the research are
∘ how these findings are communicated so they can make change happen

It might seem obvious that good research should involve the actual people that the research aims to benefit more than research participants, but this is not always the case. In order to address this, funders and organisations have specific PPIE guidelines and requirements. For early career researchers, or those whose research doesn’t usually involve direct interaction with patients or the public, it can be daunting to know how best to undertake PPIE. We’ve produced this guide to help those doing PPIE involving adults with ADHD, with tips and tricks from our experience running a range of research projects about ADHD.

## Why is it Important to Adapt PPIE Good Practice to Your Specific Groups?

PPIE isn’t just about chatting to your aunt about her experience with a health condition, and it’s also not about catching people at random on the street to ask them their thoughts on ADHD. PPIE needs to be done carefully and sensitively to help inform your project ^2^, and to make the most of what your PPIE members can input and contribute to the research process. Researchers are normally responsible for planning and leading the PPIE because they know how to plan and do research to a high standard or are going to apply for funding for a project. PPIE groups are critical in helping do the research in a way that can be meaningful and beneficial to as many people as possible. To do this well, we need insight into what it is like to live with a health condition, have specific traits, strengths or difficulties, and know what support is needed. So PPIE often involves very specific groups of people who have their own unique needs. These needs inform how the researchers go about doing PPIE. For example, if a PPIE group involves people who are having chemotherapy for cancer, you may need to have meetings at the hospital, these may need to be very short because of fatigue, or you may need to think about including people at different stages of the treatment process.

## Best Practice, Frameworks in PPIE Activities

Good practice includes PPIE activities and many, if not all, stages of the research cycle^3^. Many frameworks have been developed to support researchers in running PPIE activities^4,5^. NIHR^6^ has developed guidance and resources on the many different aspects of PPIE. From recruitment to dissemination, many considerations need to be taken to practice responsible PPIE. It is important to note that our comments/guidelines are not a PPIE framework in itself, but are in addition to these frameworks, with some adaptations and emphasis reflecting learning from working with people with ADHD.

## What are the Difficulties of Taking Part in PPIE for Adults with ADHD?

In this letter, we focus on PPIE for projects about ADHD. People with traits of ADHD can struggle with paying attention, holding onto their thoughts, or might be overactive and impulsive. Those involved in a PPIE group are separate to research participants- those who you collect research data from. This means the onus is on the researcher to make the experience ethical, positive, and engaging for your PPIE group, tailoring it to individual’s specific needs and being adaptable.

To make PPIE meetings useful and enjoyable for adults with ADHD, there are a variety of things to consider:

1. Attention spans
2. Communication preferences and how they may engage with verbal, visual or written content
3. Need for breaks, fidget moments and physical activity
4. Past experiences of formal meetings or similar events that might influence how participants feel about getting involved
5. Remembering upcoming meeting dates/times and how to access the meetings
6. Staying on track and focussing on one thing at a time

It is our responsibility to make sure we:

1. Are properly inclusive for people with ADHD
2. Run PPIE in the best way to make sure our research benefits from engagement of our group members
3. Are sensitive to individual PPIE group membership needs, and provide development and training in line with UK standards for PPIE (designed to be accessible for this group).
4. Do not inadvertently re-enforce negative experiences that people with ADHD may have already had. Key examples are negative experiences around missing meetings, forgetting to respond, or missing key information. Instead, explicitly considering (and remembering) how hard someone with ADHD works (in relation to a neurotypical brain) to perform 'routine' tasks that others may take for granted.

Bearing this in mind, we have put together specific strategies to address some of these ADHD-specific needs and considerations.

### Strategies For Planning And Running Ppie With Adults With Adhd

**Figure 1:**
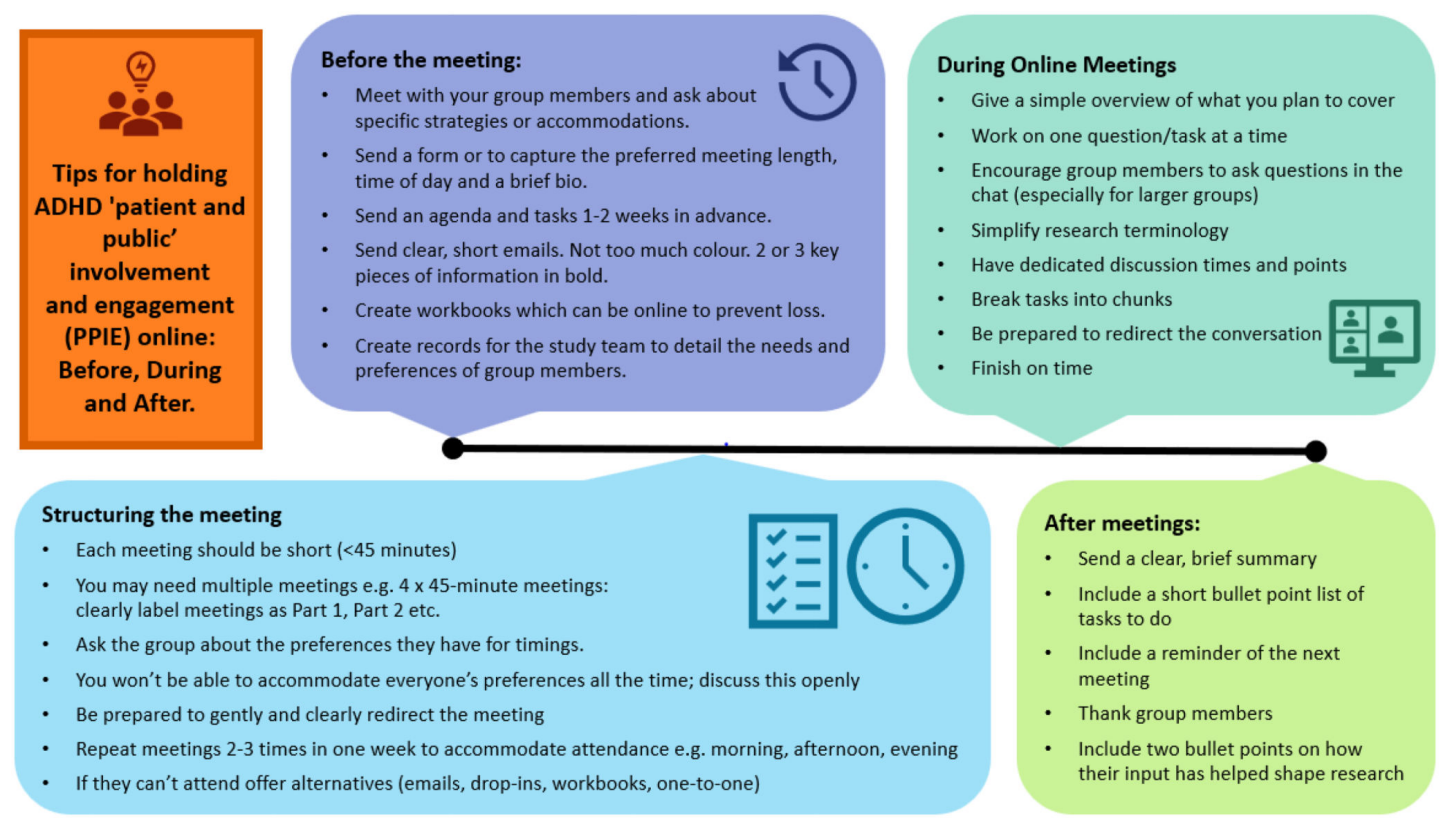
Top tips for holding online PPIE meetings with people with ADHD

Below is a list of strategies we have found help support ADHD PPIE groups. While we have found that these work, it is important to remember that ADHD is a heterogenous disorder that affects people differently and this is not a ‘one size fits all’ approach. It is important to be aware of, plan for, and account for individual differences between people with ADHD. For example, differences between type of ADHD (hyperactivity/inattention/combined), differences by gender, ethnic group, age (young people/older people/children), co-occurring ASD, physical or mental health difficulties, and past or recent life experiences. For a userfriendly infographic of these strategies please see Figure 1.

### Preparation

- Meet with potential PPIE group members individually to chat about the project and their potential involvement, and ask about any specific strategies or accommodations they may already know they need
- Send a Microsoft form or “When2meet” to capture: preferred length of meeting, preferred time of the day, brief bio, if they are happy for their email address to be shared across the PPIE group
- Send agenda and tasks for meetings to be discussed in advance (1-2 weeks)
- Send clear, short emails with bullet points for important information and bold words for dates, times, and other key information (but not too much colour or bolding as then it becomes hard to know which bits are the important ones)
- Use workbooks: It helps having all tasks are in one place, it can be picked up when they want, and you can put a link to it on OneDrive or add it to emails in case the document is lost
- Minimise text and keep same format and fonts across time
- Offer choice of incentives. Quick rewards may often be preferred (e.g. an Amazon voucher the next day rather than waiting weeks for a processed payment)
- Explore and set up preferred (and accessible) ways of communicating for group members (i.e., WhatsApp groups) and share key information (such as meeting reminders) here as well as via email. Be clear about your own professional boundaries regarding this (see below)
- Create records for the study team that detail the needs/preferences of each PPIE group member, to enable flexible and inclusive working practices

### Ground Rules In Group Meetings

- Have ground rules, agreed upon before the meeting, including:
  ∘ respecting other’s experiences and differences
  ∘ using Chat function for questions or hands up button
  ∘ no judgment
  ∘ trying not to interrupt
  ∘ being clear and specific.
- Set ground rules for group chats e.g. no politics/harassment/discrimination, to keep it a safe space for everyone

Review these in the first meeting and ask the group to add anything that would make their experience safer or better. Feedback could be anonymous so that individuals feel safe to be honest about their needs

- Be flexible with expectations: it’s ok to dip in and out within the meeting and with the PPIE process over time
- Be flexible with times. Some might text you at 10pm or want to meet early in the morning and that’s the only time they have the headspace to do this. Make your own boundaries clear as well (e.g. I will only respond to texts between 8am and 8pm, but you can send them to me whenever suits you). When defining boundaries, it is important to send a message to the whole group to avoid individuals feeling singled out and discouraged about further involvement.

### Reminders

- Send many reminders (I send 4 in 8 days), with the last one on the morning of the meeting. Be open to offering text, email or WhatsApp reminders depending on the preferences of the person
- All reminders should include the meeting link or details, each time and the software used (Teams, Zoom)
- Include the agenda

### Meetings

- Should be short, preferably not over 45 minutes.
- Ask the group about the best length of time for them. Be aware you won’t be able to accommodate everyone’s preferences all the time, and discuss this openly
- Be prepared to gently and clearly redirect during the meeting if topic goes off on a tangent. For example, you could say “this is a really interesting discussion, and I’d like us to continue it later, but right now I am going to direct us back to <this question> to ensure we get through everything that we need to in this meeting”
- Repeat meetings 2-3 times in one week to give everyone a chance and opportunity to take part (depending on capacity of the study team and the availability of group members), we often run a meeting on a weekday morning, the same meeting mid-afternoon, and a final one on Saturday mornings
- If the meeting needs to be longer than 45 minutes, have two meetings and clearly label them (and any related paperwork) as part 1 and part 2
- If they can’t attend, offer alternative options such as emails, drop-ins, one to one etc.

### During Online Meetings

- Start by providing a simple overview of what you plan to cover in that meeting
- Work on one task/question at a time
- We have found that questions from group members in the chat are easier to manage and prevent interruptions, although in small groups this may not be necessary
- Simplify research terminology/data presentation to facilitate attention
- Remind the group there are varied ways of contributing to the research. Some members may dip in and out, some may not feel comfortable using video, some may stay quiet but send comments afterwards.
- Have dedicated discussion times and points
- Break tasks into chunks and work on one chunk at a time
- Be prepared to gently redirect the meeting often. Ask people to put comments/questions in the chat if you have to move on the conversation, or send you an email or voice note with further thoughts
- Finish on time

It is useful to have two facilitators, one monitoring the chat and getting in touch with group members privately if needed, one chairing and keeping the meeting on track and to time

### After Meetings

- Send a clear, brief summary of the meeting
- Include a short bullet point lists of any tasks to do
- Include a reminder of when the next meeting will be
- Thank members and send two bullet points on ways their contributions have helped shape/direct the research
- As a researcher spend some time reflecting on how the meeting went, refining working practices for following meetings, and checking in with individual group members if needed

## Conclusion

Patient and Public Involvement and Engagement (PPIE) is a formal approach in research, particularly in the health sector, where members of the public actively participate in all stages of the research process alongside academic researchers, ensuring that research directly benefits the people it aims to serve.

PPIE is crucial but often requires adaptation to specific groups, such as adults with ADHD. Individuals with ADHD have unique needs, including attention, communication preferences, and a propensity for breaks and physical activity. Researchers must be sensitive to these needs, providing inclusive, engaging, and ethical experiences for PPIE group members.

To facilitate successful PPIE for adults with ADHD, researchers should consider their unique challenges, such as for instance difficulties in staying focused and remembering schedules and be mindful not to reinforce negative experiences, such as missed meetings or forgotten information. Instead, researchers should acknowledge the extra effort required by individuals with ADHD and develop strategies tailored to their needs.

## Lay summary

This research discusses the importance of involving individuals with ADHD in research and shares insights gained. It emphasizes the unique challenges faced by those with ADHD in terms of attention, organization, and time management, which might not always be apparent. The paper offers practical tips for researchers to better engage with ADHD groups, aiming to ensure that their experiences and perspectives are considered in the research process. In summary, it highlights the value of including firsthand experiences in ADHD research and provides guidance for researchers to enhance this inclusion.
